# Carbonized Cellulose Aerogel Derived from Waste Pomelo Peel for Rapid Hemostasis of Trauma‐Induced Bleeding

**DOI:** 10.1002/advs.202307409

**Published:** 2024-03-13

**Authors:** Wenbing Wan, Yang Feng, Jiang Tan, Huiping Zeng, Rafeek Khan Jalaludeen, Xiaoxi Zeng, Bin Zheng, Jingchun Song, Xiyue Zhang, Shixuan Chen, Jingye Pan

**Affiliations:** ^1^ The Second Affiliated Hospital, Jiangxi Medical College Nanchang University Nanchang Jiangxi 330006 China; ^2^ Key Laboratory of Intelligent Treatment and Life Support for Critical Diseases of Zhejiang Province Zhejiang Engineering Research Center for Hospital Emergency and Process Digitization The First Affiliated Hospital of Wenzhou Medical University Wenzhou Zhejiang 325000 China; ^3^ Zhejiang Engineering Research Center for Tissue Repair Materials Wenzhou Institute University of Chinese Academy of Sciences Wenzhou Zhejiang 325000 China; ^4^ Biomedical Big Data Center West China Hospital Sichuan University Chengdu China; ^5^ Wenzhou Safety (Emergency) Institute of Tianjin University Wenzhou China; ^6^ Department of Critical Care Medicine No. 908th Hospital of PLA Logistic Support Force Nanchang 330002 China; ^7^ Macau University of Science and Technology Taipa Macau 999078 China

**Keywords:** biocompatibility, carbonized pomelo peel, hemostasis, uncontrollable massive bleeding

## Abstract

Uncontrollable massive bleeding caused by trauma will cause the patient to lose a large amount of blood and drop body temperature quickly, resulting in hemorrhagic shock. This study aims to develop a hemostatic product for hemorrhage management. In this study, waste pomelo peel as raw material is chosen. It underwent processes of carbonization, purification, and freeze‐drying. The obtained carbonized pomelo peel (CPP) is hydrophilic and exhibits a porous structure (nearly 80% porosity). The water/blood absorption ratio is significantly faster than the commercial Gelfoam and has a similar water/blood absorption capacity. In addition, the CPP showed a water‐triggered shape‐recoverable ability. Moreover, the CPP shows ideal cytocompatibility and blood compatibility in vitro and favorable tissue compatibility after long terms of subcutaneous implantation. Furthermore, CPP can absorb red blood cells and fibrin. It also can absorb platelets and activate platelets, and it is capable of achieving rapid hemostasis on the rat tail amputation and hepatectomized hemorrhage model. In addition, the CPP not only can quickly stop bleeding in the rat liver‐perforation and rabbit heart uncontrolled hemorrhage models, but also promotes rat liver and rabbit heart tissue regeneration in situ. These results suggest the CPP has shown great potential for managing uncontrolled hemorrhage.

## Graphical abstract

In this study, waste pomelo peel is chosen as raw material to produce a hemostatic sponge. It underwent processes of carbonization, purification, and freeze‐drying. The obtained carbonized pomelo peel (CPP) is hydrophilic, and it exhibits a porous structure and effective hemostatic properties.

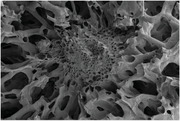

## 1 Introduction

The human blood coagulation mechanism can usually stop bleeding caused by some small wounds, while it does not work in uncontrollable massive bleeding that mainly occurs in the abdominal cavity and limbs.^[^ ^1^ ^]^ Severe bleeding affects normal blood circulation, leading to blood microcirculation disorder that can introduce ischemia and hypoxia in various organs.^[^ ^2^ ^]^ Moreover, it may induce more serious complications, such as hemorrhagic shock, organ failure, hypothermia, hypotension, and even death.^[^ ^3^ ^]^ According to relevant research surveys, massive bleeding accounts for 35% of trauma mortality, 40% of which occurs before admission.^[^ ^4^ ^]^ Most hemorrhagic deaths occur within the 1 h of bleeding. Therefore, it is necessary to develop hemostatic materials that can achieve rapid hemostasis for abdominal cavity and limb hemorrhage application. It can provide enough transfer time for the injured to the hospital for surgery.

Our study aims to develop hemostatic materials using naturally derived biomaterials due to their good hydrophilicity and abundant supply of raw materials. Naturally derived biomaterials such as gelatin,^[^ ^5^ ^]^ bioglass,^[^ ^6^ ^]^ zeolite,^[^ ^7^ ^]^ alginate, chitosan, cellulose,^[^ ^8^ ^]^ and collagen^[^ ^9^ ^]^ have been widely used for developing hemostatic materials. Many forms of hemostatic materials, for example, electrospun nanofibers, hydrogels,^[^ ^10^ ^]^ sponges,^[^ ^5^, ^11^ ^]^ micro/nanoparticles,^[^ ^12^ ^]^ non‐woven fabrics,^[^ ^13^ ^]^ and microneedles^[^ ^14^ ^]^ have been developed and made some achievements. Among these hemostatic materials, hemostatic sponges have the most advantages, because they have high blood absorption capacity and fast speed of blood absorption and concentration. In our previous study, we developed a carbonized cellulose‐based aerogel for the management of noncompressible torso hemorrhage.^[^ ^15^ ^]^ The carbonized cellulose aerogels show desirable good blood absorption and coagulation ability both in vitro and in vivo. However, the porosity of carbonized cellulose aerogels is not high enough, and the pores are not connected. Porosity and interpenetrating pore structure are critical in blood absorption and coagulation.^[^ ^16^ ^]^

To address the problems existing in the internal structure of our developed carbonized cellulose aerogel, one feasible approach is to find new cellulose raw materials with high porosity and interconnected pores. By chance, we found the waste pomelo peel had a naturally interpenetrating porous structure, which can be obtained in large quantities. In addition, the main component of pomelo peel is cellulose. It is also applicable to the carbonization method we developed previously. We hypothesize that using pomelo peel as raw material to make carbonized cellulose aerogel without destroying its original structure, and use it to stop massive bleeding quickly. In this study, we will study the impact of the carbonization treatment process on aerogel's structure, biocompatibility, and blood‐absorbing and coagulation properties in vitro. We will also explore the hemostatic ability of the carbonized pomelo peel in four different hemorrhage models.

## 2 Results and Discussion

### 2.1 Preparation and Characterization of CPP

**Figure 1:**
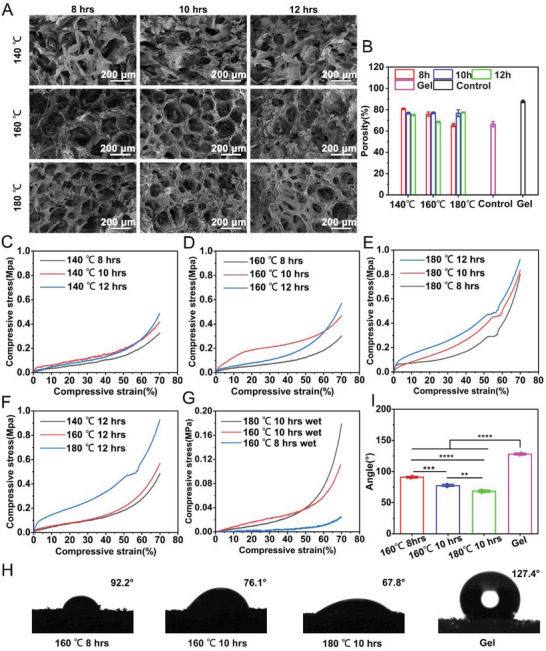
The physical characterization of carbonized pomelo peel sponge (CPP). A) SEM images show the internal structure of CPP after hydration thermal reaction at 140 °C, 160 °C, and 180 °C for 8, 10, and 12 h, respectively. B) Porosity quantification of different types of CPP, the gelfoam (Gel) as a positive control. C) The compression strain‐stress curve of different CPPs that carbonized at 140 °C for 8, 10, and 12 h, respectively. D) The compression strain‐stress curve of different CPPs that carbonized at 160 °C for 8, 10, and 12 h, respectively. E) The compression strain‐stress curve of different CPPs that carbonized at 180 °C for 8, 10, and 12 h, respectively. F) The compression strain‐stress curve of different CPPs that carbonized at 140 °C, 160 °C, and 180 °C for 12 h, respectively. G) The compression strain‐stress curve of (160 °C, 8 h) CPP, (160 °C, 10 h) CPP, and (180 °C, 10 h) CPP under wet conditions. H) The surface contact angle of (160°C, 8 h) CPP, (160 °C, 10 h) CPP, (180 °C, 10 h) CPP, and gelfoam. I) Quantification of the contact angle of different CPPs and gelfoam. **p <0.01, ***p <0.001, ****p <0.0001.

The internal structure of natural pomelo peel presents an interconnected porous structure (Figure S1, Supporting Information), which plays an essential role in blood uptake and concentration.^[^ ^17^ ^]^ However, the natural pomelo peel contains flavonoids, polyphenols, and other bioactive molecules,^[^ ^18^ ^]^ which may enter the blood circulation system after the hemostasis process and will impact people's health. To remove these bioactive substances, we carbonize the natural pomelo peel. The main component of pomelo peel after carbonization is carbon‐based materials. The carbonized pomelo peel (CPP) aerogel was prepared by a one‐pot hydrothermal reaction.^[^ ^15^ ^]^ **Figure** **1**A shows the internal porous structure of the CPP aerogels. The CPP aerogels' internal structure that carboned at 140 °C for 8, 10, and 12 h collapsed after carbonization. Similarly, the CPP aerogels (160 °C, 12 h; 180 °C, 12 h) structures collapsed after carbonization. The final structure of CPP aerogels (160 °C, 8 and 10 h; 180 °C, 8 and 10 h) was close to the original porous structure. The porosity of original pomelo peel was ≈66% (Control) (Figure 1B). The porosity of CPPs was measured by the ethanol substitution method.^[^ ^19^ ^]^ The CPPs' porosity was nearly 80%, which was slightly lower than the gelfoam (≈87%, positive control) (Figure 1B).

The hemostatic material should have certain mechanical properties to cooperate with pressing to stop bleeding.^[^ ^20^ ^]^ The compression tests revealed that the compressive properties and Young's modulus of the CPPs had a growing trend with the increase of carbonization time at the same carbonization temperature (Figure 1C–E; Figure S2A, Supporting Information). In addition, the compressive properties and Young's modulus of the CPPs with the same carbonization time increased gradually with the increase of carbonization temperature (Figure 1F). We also detected the compressive properties of CPPs under wet conditions. As shown in Figure S2B–D (Supporting Information) and Figure 1G, the compressive properties and Young's modulus of the CPP aerogels after wetting were significantly reduced compared to those in dry conditions. Besides compression tests, we also explored the tensile properties of the CPPs. The tensile modulus of the CPPs with the same carbonization temperature gradually increased with the increase of carbonization time (Figure S3, Supporting Information).

Finally, we checked the hydrophilicity of the CPPs after carbonization. The contact angles of CPP aerogels (160 °C, 8 and 10 h; 180 °C, 10 h) were smaller than gelfoam. Especially in the (160 °C, 10 h) CPP aerogel and (180 °C, 10 h) CPP aerogel (Figure 1H,I), the contact angle was 76.1° and 67.8°, suggesting the CPPs were hydrophilic materials, which was conducive to blood absorption concentration.^[^ ^16^, ^21^ ^]^

### 2.2 The Swelling Behaviors of CPP

**Figure 2:**
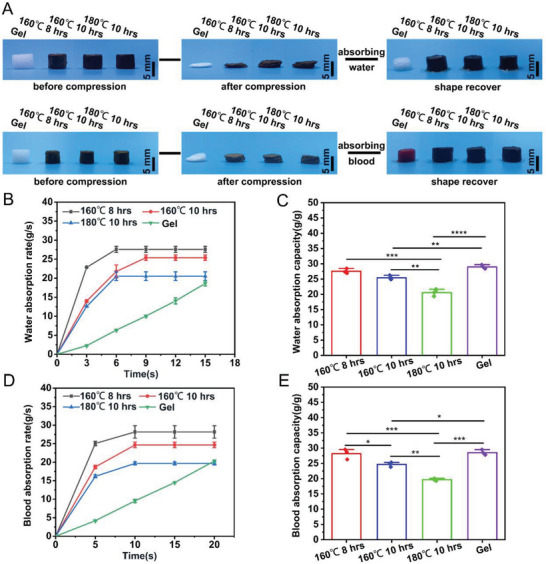
The swelling and shape recoverable behaviors of CPP. A) Photographs visualize the hydration‐induced shape recovery of various CPPs and gelfoam before and after compression in water or blood. B) The water absorption rate of various CPPs and gelfoam over time. C) The maximum water absorption capacity of various CPPs and gelfoam. D) The blood absorption rate of various CPPs and gelfoam over time. E) The maximum blood absorption capacity of various CPPs and gelfoam. *p <0.05, **p <0.01, ***p <0.001, ****p <0.0001.

Hemostatic materials used for non‐compressible torso hemorrhage are recommended to have the shape recoverable ability after compression, so that the volume of the hemostatic material can be significantly compressed, making it easier to deliver it into the abdominal cavity for hemostasis.^[^ ^17^, ^22^ ^]^ Therefore, we examined the shape recovery ability of CPPs. Three types of CPP aerogel (160 °C, 8 and 10 h; 180 °C, 10 h) and gelfoam were placed on a plastic substrate and compressed, then DI water and whole blood were added, and the deformation recovery ability of the samples was recorded. After adding water and blood, we found the three types of CPP aerogel and gelfoam could fully restore their original shape (**Figure** **2**A; Figure S4, Supporting Information).

Rapid hemostasis usually requires hemostatic materials to absorb blood from excessive bleeding wounds quickly, so we compared the water and blood absorption ability of the CPPs.^[^ ^23^ ^]^ As shown in Figure 2B, the water absorption speed of CPP aerogel was significantly faster than the gelfoam. The three types of CPP aerogels could reach swelling equilibrium within 3–6 seconds, while the gelfoam needed ≈18 s. The final total water absorption capacity of CPP aerogel (160 °C, 8 h), CPP aerogel (160 °C, 10 h), CPP aerogel (180 °C, 10 h), and gelfoam were (27.57 ± 0.46), (25.38 ± 0.42), (20.53 ± 0.66), (28.97 ± 0.38) g g^−1^ (Figure 2C). In addition, the CPP exhibited a similar fast blood absorption speed compared to the gelfoam (Figure 2D). The final blood absorption of CPP aerogel (160 °C, 8 h), CPP aerogel (160 °C, 10 h), CPP aerogel (180 °C, 10 h), and gelfoam were (28.17 ± 0.97), (24.67 ± 0.44), (19.67 ± 0.23), (28.53 ± 0.50) g g^−1^ (Figure 2E). The commercialized gelfoam as the positive control in this study, the presented CPPs (160 °C, 8 and 10 h) exhibit faster water/blood absorption ratio and similar water/blood absorption capacity.

### 2.3 Biocompatibility of the CPP

**Figure 3:**
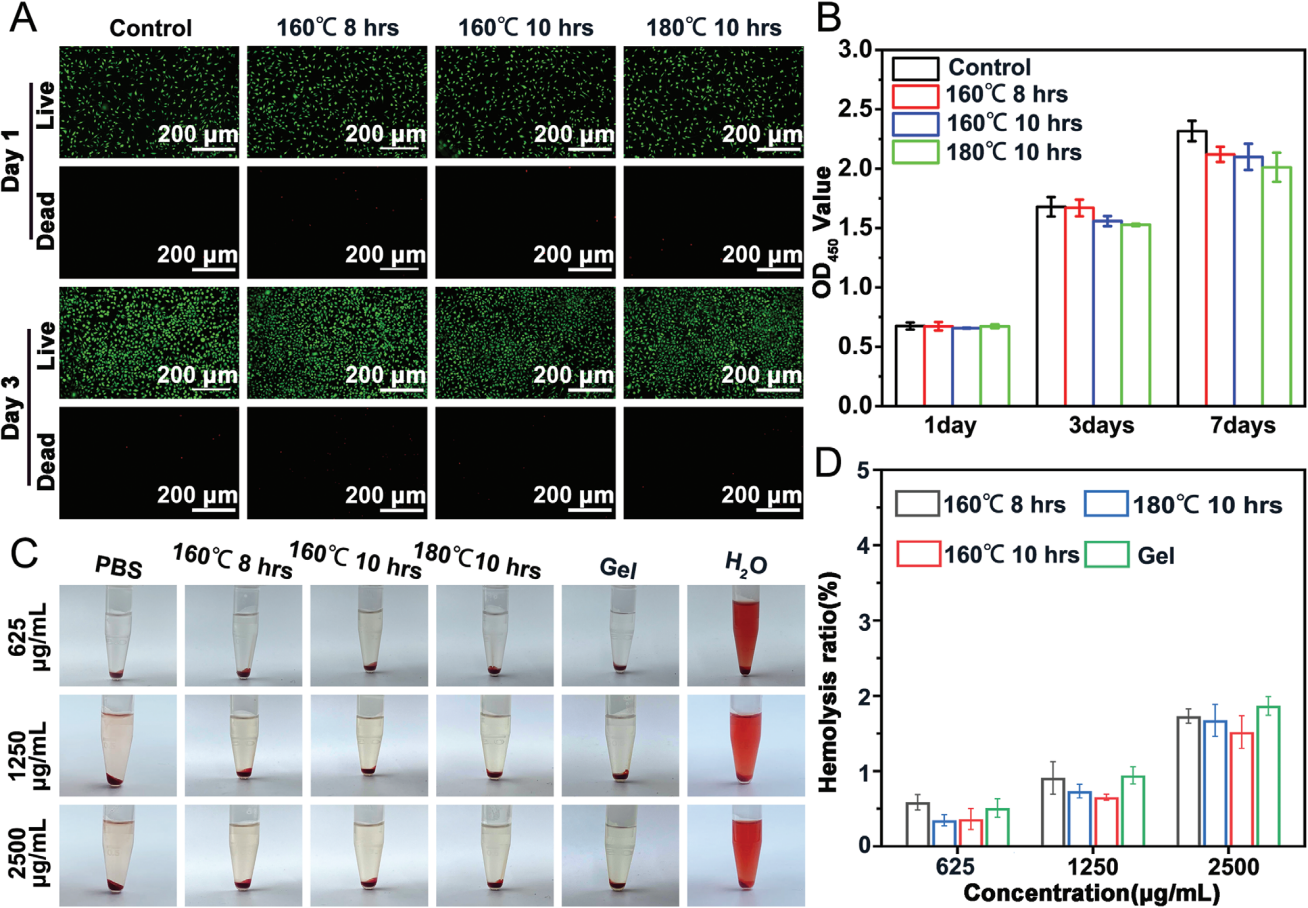
In vitro biocompatibility of CPP. A) Live/Dead staining of L929 cells treated with different CPP extracts for 1 and 3 days. B) The proliferation of L929 cells co‐cultured with different CPP extracts for 1, 3, and 7 days. C) In vitro hemolysis test of different CPPs and gelfoam with 625, 1250, and 2500 µg concentrations, respectively. D) Quantitative analysis of hemolysis test of different CPPs and gelfoam.

Since hemostatic materials are in direct contact with the blood circulatory system, high biocompatibility is essential for hemostatic materials.^[^ ^24^ ^]^ First, the extracts of the CPP were prepared to examine its cytocompatibility. As shown in **Figure** **3**A, the live/dead staining revealed that the extracts of the CPP (160 °C, 8 and 10 h; 180 °C, 10 h) there was no apparent cytotoxicity on L929 cells after 1 and 3 days of co‐culture. L929 cells gradually increased from 1 to day 7 (Figure 3B). In addition, the hemolysis test discovered that the CPP also has good blood compatibility (Figure 3C,D). We selected three concentrations, 625, 1250, and 2500 µg mL^−1^ of different CPPs to evaluate blood compatibility. The results showed that the aerogel group was light red, similar to the negative control (PBS group) and positive control (gelfoam group). In contrast, the water‐treated group showed obvious hemolysis (Figure 3C). The quantitative results showed that the hemolysis rate of each concentration of aerogel was lower than 5% (Figure 3D), indicating that the aerogel has good blood compatibility.

**Figure 4:**
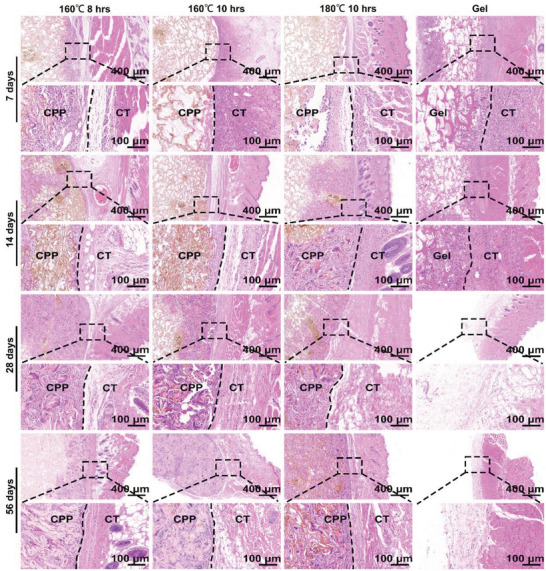
The tissue compatibility of different CPPs. H&E staining images of different CPPs and gelfoam after subcutaneous implantation for 7, 14, 28, and 56 days. CT: connective tissue, CPP: carbonized pomelo peel.

Besides the in vitro studies, long‐term subcutaneous implantation experiments were performed to test the tissue compatibility of CPPs. The generation of fibrous capsules and the thickness of the fibrous capsules are the most intuitive indicators for evaluating the tissue compatibility of materials.^[^ ^25^ ^]^ As shown in **Figure** **4**, S5 (Supporting Information), although there was little cell infiltration in the CPP groups compared to the gelfoam group, no significant fibrous capsules formed in all CPP groups after 7 days of implantation. The fibrous capsule formed in the gelfoam group had a higher cell density than its surrounding tissues. After 14 days of implantation, improved cell infiltration was observed in all CPP groups, but there are still areas with no cell infiltration. And there are also areas without cell infiltration within the gelfoam group. After 28 days of implantation, the gelfoam has completely degraded. And the cell infiltrated areas of these CPP groups were significantly increased compared to it on day 14. After 56 days of implantation, the three types of CPP were entirely filled with infiltrated cells, and the formed granulation tissues were close to normal dermis tissue. However, the CPP didn't completely degrade after 56 days of implantation. To further evaluate its long‐term tissue compatibility, we further explored the infiltration of macrophages, the main inflammatory cells among the four groups after subcutaneous implantation. As shown in Figures S6 and S7 (Supporting Information), the infiltrated macrophages (pan marker: CD68 and F4/80) of the gelfoam group were significantly lower than the three CPPs groups after 14 days of implantation. In addition, the macrophage infiltration gradually decreased in the three CPP groups from 7 to day 56. Suggesting the inflammatory response caused by CPPs gradually decreases after long‐term implantation, the CPPs were gradually accepted by the host.

### 2.4 In Vitro Hemostatic Properties of CPP Aerogels

**Figure 5:**
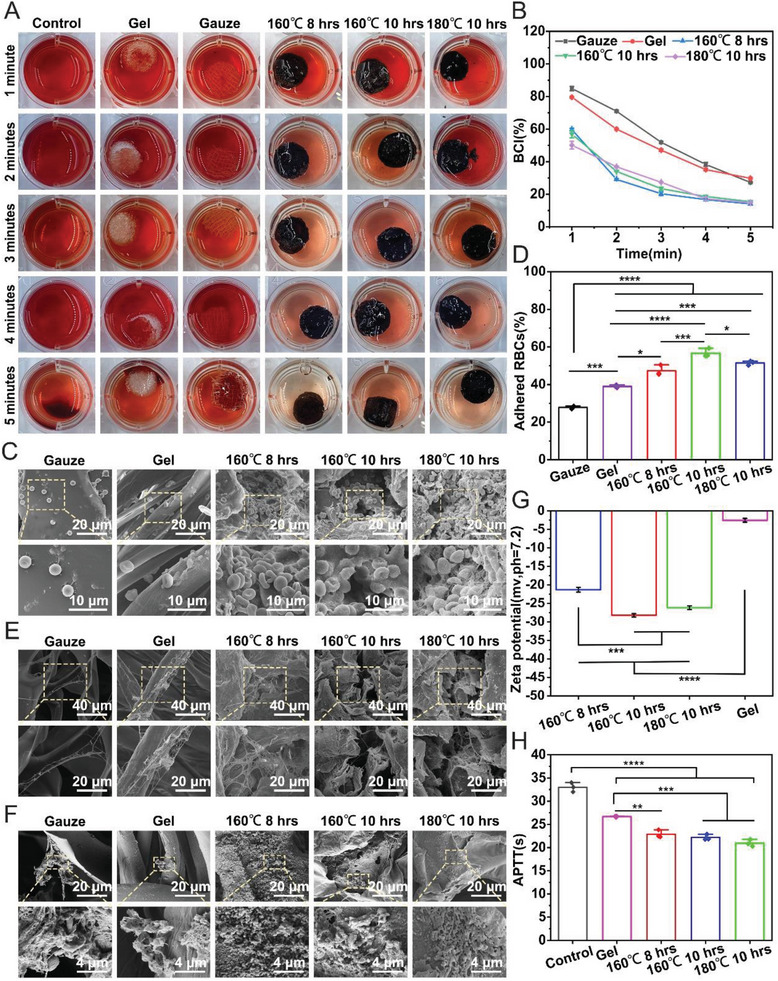
The in vitro hemostatic ability of CPPs. A) Hemoglobin binding capacity of various CPPs, gauze, and gelfoam at 1, 2, 3, 4, and 5 min. B) Blood clotting index (BCI) (%) of different CPPs, gauze, and gelfoam across detection time points. C) SEM images exhibiting RBC absorption on the surface of different CPPs, gauze, and gelfoam. D) Percentage of adhered RBCs on different CPPs, gauze, and gelfoam surfaces. E) SEM visualization of fibrin adhesion on different CPPs, gauze, and gelfoam surfaces. F) SEM images showing platelet adhesion on different CPPs, gauze, and gelfoam surfaces. G) Zeta potential of the different CPPs and gelfoam sponge. H) Activated partial thromboplastin time (APTT) analysis of the different CPPs and gelfoam. *p <0.05, ***p <0.001, ****p <0.0001.

The in vitro coagulation capacity was assessed by whole blood clotting assays. Aerogels were placed in 24‐well plates, and 100 µL of recalcified blood was rapidly pipetted onto each sample surface. Plates were incubated at 37 °C for 1, 2, 3, 4, and 5 min. Then, 2 mL DI water was added to lyse unclotted erythrocytes. The control, gauze, and gelform groups appeared fresh red, indicating fewer trapped red blood cells within clots. In contrast, aerogels maintained a lighter red color, demonstrating superior capture of erythrocytes into localized clots (**Figure** **5**A). The blood clotting index (BCI) (%) of different CPPs was significantly lower than the gauze and gelfoam groups at each indicated time (Figure 5B). We utilized SEM to visualize interactions between the materials and blood components to elucidate the hemostatic mechanism further. We first examined erythrocyte adhesion, finding robust attachment in the aerogel group versus minimal bonding in the gelfoam and gauze groups (Figure 5C,D). Additionally, abundant fibrin network formation occurred on aerogels, contrasting the sparse fibrin observed on gelfoam and gauze (Figure 5E). Finally, aerogels exhibited greater platelet adhesion and activation versus the other groups, although no significant differences existed among the aerogel samples (Figure 5F).

We also explored the potential pro‐coagulant mechanism. The coagulation performance of hemostatic materials is related to its charges.^[^ ^26^ ^]^ Thus, we tested the zeta potential of CPPs. The zeta potential of the (160 °C 8 h) CPP, (160 °C 10 h) CPP, and (180 °C 10 h) CPP was(−21.3 ± 0.63), (−26.17 ± 0.50), and (−28.23 ± 0.47) mV respectively, which was significantly lower than the zeta potential of gelfoam (−2.54 ± 0.51) mV (Figure 5G). The negative charges are capable of accelerating intrinsic blood coagulation by activating prekallikrein, cofactors HWK‐kininogen, and coagulation factors XI and XII, resulting in a fast coagulation.^[^ ^27^ ^]^ We measured the partial activated thromboplastin time (APTT), there was no significant difference in the APTT among the CPP groups, but it was lower than the blank and gelfoam groups (Figure 5H). We speculate that the rapid coagulation mechanism of CPPs is related to its high negative charge, which is similar to our previously developed carbonized cellulose aerogels.^[^ ^15^ ^]^

### 2.5 In Vivo Hemostatic Ability of CPPs

**Figure 6:**
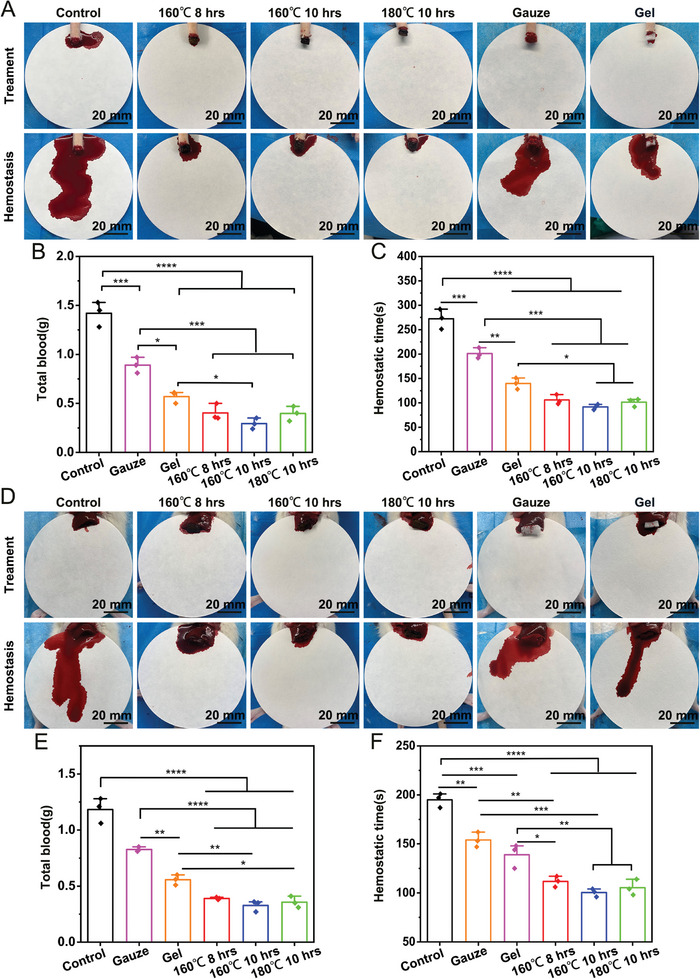
The performance of the carbonized CPPs on the rat tail amputation and hepatectomized hemorrhage model. A) Photographs showing the hemostatic ability of different CPPs, gauze, and gelfoam on the rat tail amputation hemorrhage model. B,C) Quantitative analysis of the total blood loss and hemostatic time of different CPPs, gauze, and gelfoam on the rat tail amputation hemorrhage model. D) Photographs showing the hemostatic ability of different CPPs, gauze, and gelfoam on the rat hepatectomized hemorrhage model. E,F) Quantitative analysis of the total blood loss and hemostatic time of different CPPs, gauze, and gelfoam on the rat hepatectomized hemorrhage model. *p <0.05, **p <0.01, ***p <0.001, ****p <0.0001.

First, we used the rat tail amputation bleeding model to test the hemostatic ability of the CPP aerogels (**Figure** **6**A). The total blood loss of the (160 °C, 8 h) CPP, (160 °C,10 h) CPP, and (180 °C, 10 h) CPP were (0.403 ± 0.048) g, (0.293 ± 0.032) g, and (0.397 ± 0.043) g, respectively. The total blood loss in the three CPP‐treated groups was significantly lower than in the gauze (0.890 ± 0.046) g and control (1.420 ± 0.074) g groups. And the total blood loss of (160 °C,10 h) CPP was reduced compared to the gelfoam group (0.57 ± 0.035) g (Figure 6B). We also measured the coagulation time, and it can be seen that the (160 °C,10 h) CPP showed the fastest coagulation time (91.67 ± 5.51) s, which was much lower than the gelfoam group (139.67 ± 11.50) s and the gauze group (201 ± 10.82) s (Figure 6C). Following, we examined the hemostatic ability of CPPs on the rat hepatectomized hemorrhage model (Figure 6D). Similarly, the rats treated with CPP aerogels had the lowest blood loss (Figure 6E) and fastest hemostatic time (Figure 6F) compared to gauze and gelfoam.

**Figure 7:**
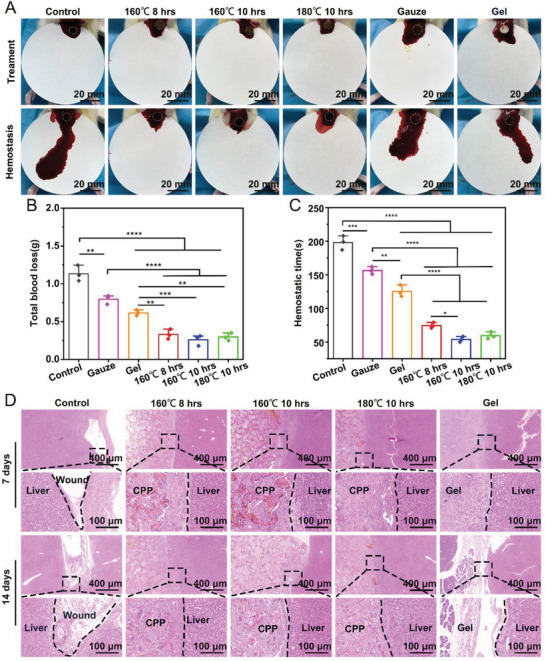
The hemostasis and tissue repair ability of CPPs on the rat liver‐perforation hemorrhage model. A) Photographs showing the hemostatic ability of different CPPs, gauze, and gelfoam on the rat liver‐perforation hemorrhage model. B,C) Quantitative analysis of the total blood loss and hemostatic time of different CPPs, gauze, and gelfoam on the rat liver‐perforation hemorrhage model. D) H&E staining images exhibit the interface between liver and CPP, gauze, and gelfoam after 7 and 14 days of treatment.

**Figure 8:**
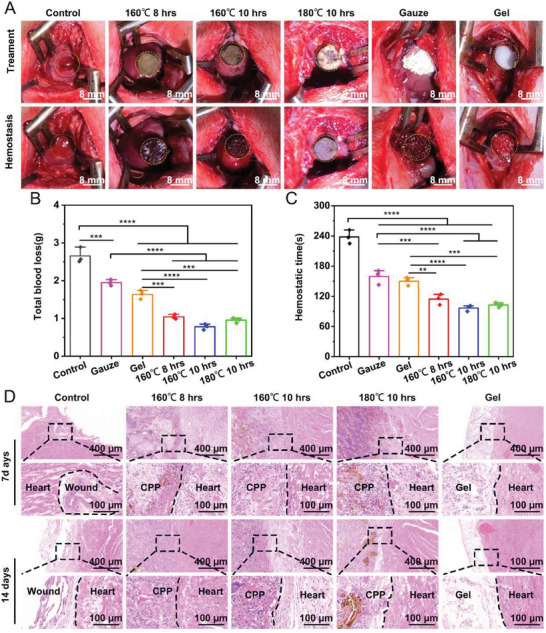
The hemostasis and tissue repair ability of CPPs on the rabbit heart uncontrolled hemorrhage model. A) Photographs showing the hemostatic ability of different CPPs, gauze, and gelfoam on the rabbit heart uncontrolled hemorrhage model. B,C) Quantitative analysis of the total blood loss and hemostatic time of different CPPs, gauze, and gelfoam on the rabbit heart uncontrolled hemorrhage model. D) H&E staining images exhibit the interface between heart and CPP, gauze, and gelfoam after 7 and 14 days of treatment.

In previous studies, we only briefly evaluated the hemostatic effect of the hemostatic material, but the pro‐healing effects on damaged organs were unclear. In this project, we assessed CPP's hemostasis and tissue repair ability in the rat liver‐perforation and rabbit cardiac perforation hemorrhage models. As shown in **Figure** **7**A–C, in the rat liver‐perforation hemorrhage model, the bleeding volume of each CPP aerogel group was (0.333 ± 0.07) g, (0.26 ± 0.07) g, (0.297 ± 0.05) g, which was lower than that of gelfoam (0.614 ± 0.04) g, gauze(0.796 ± 0.06) g, and blank group (1.13 ± 0.11) g. In addition, the coagulation time of the CPPs was (74.33 ± 4.16) s, (53.67 ± 4.04) s, (59.33 ± 5.13) s, which were faster than gelfoam (125.33 ± 8.74) s, gauze (156.33 ± 5.50) s, and the blank group (198 ± 10.54) s. Moreover, the injured liver tissue was regenerated 7 and 14 days after hemostasis compared to the control and gelfoam groups (Figure 7D). In the rabbit cardiac perforation hemorrhage model, the bleeding volume of each CPP‐treated group was (1.043 ± 0.035) g, (0.780 ± 0.043) g, (0.953 ± 0.038) g, which was lower than that of gelfoam (1.633 ± 0.067) g, gauze (1.947 ± 0.046) g, and blank group (2.657 ± 0.118) g. In addition, the coagulation time of the aerogel was (114.33 ± 6.12)s, (96.47 ± 3.38) s, (102.67 ± 2.60) s, which were faster than gelatin sponge (150.00 ± 4.73) s, gauze (159.33 ± 8.41) s, and the blank group (238.00 ± 7.81) s (**Figure** **8**A–C). The injured heart tissue in the control group still obviously showed wounded status, while the CPPs and gelfoam treated heart tissue were protected by formed fibrosis tissues (Figure 8D).

## 3 Conclusion

In this study, we developed a new carbonized cellulose aerogel based on pomelo peel for hemostasis application. It underwent processes of carbonization, purification, and freeze‐drying. The obtained carbonized pomelo peel (CPP) was hydrophilic and exhibited a porous structure (nearly 80% porosity). The water/blood absorption ratio was significantly faster than the commercial Gelfoam and had a similar water/blood absorption capacity. In addition, The CPP showed a water‐triggered shape‐recoverable ability. Moreover, the CPP showed ideal cytocompatibility and blood compatibility in vitro and favorable tissue compatibility after long terms of subcutaneous implantation. Furthermore, CPP could absorb red blood cells and fibrin. It also could absorb platelets and activate platelets in vitro. In vivo, the CPP is capable of achieving rapid hemostasis on the rat tail amputation and hepatectomized hemorrhage model. In addition, the CPP not only could quickly stop bleeding in the rat liver‐perforation and rabbit heart uncontrolled hemorrhage models, but also promote rat liver and rabbit heart tissue regeneration in situ. The presented carbonized pomelo peel aerogels show great promising for the management of uncontrolled bleeding.

## 4 Experimental Section

### Materials

The pomelo peel used in the experiment was purchased from a store (Jiangyong Xiangyou, Yongzhou). Gelfoam was ordered from Quick Health Medical Technology(catalog number: 2640494, Guangzhou, China). Tris‐HCl was purchased from GBCBIO Biotechnology company (catalog number: T0688, Guangzhou, China). Dulbecco's Modified of Eagle's Medium (DMEM medium) (catalog number: 11965118), and penicillin‐streptomycin (PS, catalog number: 15140148) mixture were purchased from Solarbio Biotechnology company (Beijing, China), and fetal bovine serum (FBS catalog number: 10099–141C) was purchased from Cellmax Cell Technology company (Suzhou, China). Trypsin‐EDTA was purchased from GBCBIO Biotechnology company (catalog number: 0322, Guangzhou, China), live cell/dead cell staining kit was purchased from bestbio company, and cell counting kit‐8 (CCK‐8) was purchased from APEXBIO Biotechnology company (catalog number: CO040, Suzhou, China). The activated partial thromboplastin time kit (APTT) was purchased from Shanghai Sun Biotechnology company (catalog number: ZY602874B, Shanghai, China), and the universal tissue fixative was purchased from GBCBIO Biotechnology company (catalog number: G0528, Guangzhou, China). Anhydrous ethanol was purchased from Sinopharm chemical (catalog number: 10 009 228, Beijing, China). All remaining reagents were purchased from Aladdin Technology company.

### Preparation of the CPP

The CPP aerogels were prepared by hydrothermal method. Pomelo peel was decorticated, cut into sections, and added to Teflon‐lined stainless steel autoclave reactor. Hydrothermal reactions were performed at 140, 160, or 180 °C for 8, 10, or 12 h, respectively. After cooling the reactor to room temperature, the aerogel products were sequentially immersed in absolute ethanol and deionized water to purify the aerogels, and then freeze‐drying all samples at −20 °C for 24 h.

### Physical Characterization of the CPP

Scanning electron microscopy (SEM) was used to observe the internal structure of each sample. The zeta potential of the CPP aerogels was determined by Nano Particle Size and Zeta Potential Analyzer. The surface tension/contact angle measuring instrument was used to obtain the water contact angle of the aerogel surface. Here, the method of detecting porosity was wanted to specified. The porosity of the cellulose aerogels was determined using the ethanol displacement method. The aerogels' initial dry weight (Ws) and volume (V) were measured. The dried aerogels were fully immersed in absolute ethanol until they reached saturation. Residual liquid on the sample surfaces was wicked away with filter paper before weighing the soaked aerogel mass (Wt). Porosity was calculated using the following equation: Porosity (%) = (W_t_‐W_s_)/ρV × 100%. Where Wt is the weight of the aerogel after soaking in absolute ethanol, Ws is the weight of the dry aerogel, V is the volume of the dry aerogel, and ρ is the density of absolute ethanol (0.7893 g cm^−3^).

### Swelling Behavior of the CPP

The CPP aerogels (5 mm in height, 8 mm in diameter) were immersed in deionized water. The weight was recorded at 0, 3, 6, 9, 12, and 15 seconds, respectively. The gelfoam was used as control. The water absorption capacity was calculated using the equation:

(1) $$\mathrm{Water}/\mathrm{blood}\;\mathrm{absorption}\;\mathrm{capacity}\left(g/g\right):\left(W_{2}-W_{1}\right)/W_{2}$$

(2) $$\mathrm{Water}/\mathrm{blood}\;\mathrm{absorption}\;\mathrm{rate}\;\left(g/s\right):\left(W_{2}-W_{1}\right)/\mathrm{time}$$

where W_2_ is the weight after immersion in deionized water/whole blood at different times, and W_1_ is the weight of the dry aerogel.

### Mechanical Tests

The compression and tensile tests of the CPP aerogels (5 mm in height, 8 mm in diameter) were performed by an electromechanical general testing machine. In the compression experiment, the dry aerogels and wet aerogels were placed on the instrument table, then samples were compressed at a constant rate to 70% strain. Similarly, the stretching experiment was performed. The dry CPPs were placed on an instrument, stretching the sample constantly until it broke.

### Cytotoxicity Experiments

L929 cells were placed in DMEM medium containing 10% fetal bovine serum and 1% penicillin‐streptomycin, cultured at 37 °C, 5% CO2, and cytotoxicity of CPPs were performed by using CCK‐8 and Calcein‐AM/PI double staining. The CPPs were sterilized under UV light for 24 h, then soaked in the complete growth medium in a 100 mg/5 mL ratio for 72 h. L929 was seeded in 96‐well plates at a density of 4000 cells per well. After 24 h of culture, the medium was replaced with CPPs’ extract medium and continuously cultured for 1, 3, and 5 days. At each indicated time point, 10 uL CCK‐8 solution was added per well and incubated for 2 h at 37 °C. The absorbance value at 450 nm was detected with a microplate reader. In addition, the Calcein‐AM/PI double staining was used to further detect the biocompatibility of CPPs. Briefly, L929 cells were seeded in 96‐well plate with a 4000 pcs/well density. The culture medium was replaced with CPPs extract medium after 24 h of culture. Then cells were continued to culture for 1 and 3 days, and then stained with AO/EB for 30 min, and then immediately observed the L929 cells with a microscope.

### Hemolysis Experiments

Sodium citrate‐anticoagulated whole blood was centrifuged at 3000 rpm for 10 min. The supernatant was removed, and erythrocytes were washed three times with PBS. A 5% RBC suspension was prepared by mixing 0.5 mL RBCs with 9.5 mL PBS. Cellulose aerogels and gelatin sponges were frozen in liquid nitrogen, pulverized into powders, and dissolved in PBS at 625, 1250, and 2500 µg mL^−1^. 0.5 mL sample suspensions were mixed with 0.5 mL 5% RBC suspension and incubated at 37 °C for 1 h. Tubes were centrifuged at 1000 rpm for 10 min. Supernatants were transferred to a 96‐well plate, and absorbance was measured at 562 nm. DI water and PBS served as positive and negative controls, respectively. Hemolysis was calculated as follows:

(3) $$\mathrm{Hemolysis}\;\mathrm{rate}\left(\%\right)=\left(OD_{s}-OD_{p}\right)/\left(OD_{h}-OD_{p}\right)\times 100\%$$

where OD_s_ is the absorbance value of the sample, OD_p_ is the negative control PBS absorbance value, and OD_h_ is the positive control deionized water absorbance value.

### In Vitro Coagulation Experiment

Cellulose aerogel, gelfoam, and gauze samples (5 mm x 8 mm) were arranged in 24‐well plates. 1 mL 0.1% calcium chloride was mixed with 9 mL sodium citrate‐anticoagulated whole blood. 100 µL of recalcified blood was rapidly pipetted onto each sample surface. After incubating at 37 °C for 1, 2, 3, 4, and 5 min, 2 mL DI water was added to lyse unclotted erythrocytes without disrupting clot formation. Supernatants were collected in 96‐well plates and absorbance was read at 562 nm. 100 µL whole blood mixed with 2 mL DI water served as a control. The blood clotting index (BCI) was calculated as:

(4) $$\mathrm{BCI}\left(\%\right)=\left(OD_{2}/OD_{1}\right)\times 100\%$$

where OD_2_ is the absorbance value of the sample, and OD_1_ is the absorbance value of the control group at 0 min.

### Red Blood Cell and Platelet Adhesion Experiments

CPP aerogels, gelfoam, and gauze samples (5 mm x 8 mm) were arranged in 24‐well plates. 100 µL whole blood was pipetted onto each sample surface and incubated at 37 °C for 5 min. Samples were washed 3x with PBS to remove non‐adherent erythrocytes. 2 mL DI water was added to lyse attached RBCs, plates were mixed well, and supernatants were transferred to 96‐well plates for absorbance reading at 562 nm. 100 µL whole blood mixed with 2 mL DI water served as a control. The percentage of RBC adhesion was calculated as:

(5) $$\mathrm{Red}\;\mathrm{blood}\;\mathrm{cell}\;\mathrm{adhesion}\;\mathrm{rate}\left(\%\right)=\left(A_{s}/A_{h}\right)\times 100\%$$

where A_s_ is the absorbance of the sample and A_h_ is the absorbance of control group.

For erythrocyte adhesion, 100 µL whole blood was pipetted onto each sample surface and incubated at 37 °C for 1, 2, or 3 min. Samples were then washed three times with PBS, fixed in 4% paraformaldehyde for 2 h, dehydrated in ethanol, and freeze‐dried for SEM imaging. To visualize platelet adhesion, 50 µL PRP was pipetted onto cellulose aerogel, gelatin sponge, and gauze surfaces, followed by 37 °C incubation for 30 min. Samples were washed 3x with PBS, fixed in 4% paraformaldehyde for 2 h, dehydrated in ethanol, and freeze‐dried for SEM imaging.

### APTT Assay

Platelet‐free plasma was isolated by centrifuging sodium citrate‐anticoagulated whole blood at 3000 rpm for 10 min following the activated partial thromboplastin time kit protocol. All reagents and materials were pre‐warmed to 37 °C before testing. CPP aerogels and gelfoam were mixed with 1 mL platelet‐free plasma, then equal volumes of 0.025 m CaCl_2_ were added to initiate clotting. Finally, the clotting times were recorded.

### In Vivo Hemostasis Experiments

All animal experiments were licensed by the Animal Ethics and Welfare Center of Nanchang University (NCULAE‐20221031136). All rats were purchased from Tianqin Biotechnology Company and rabbits from Ganzhou Animal Husbandry and Fisheries Research Institute. The hemostatic performance of cellulose aerogels was evaluated rat tail amputation, hepatectomy, liver‐perforation hemorrhage model, and rabbit heart uncontrolled hemorrhage model.

In the rat tail amputation hemorrhage model, the rats were randomly divided into 6 groups (n = 3). First, the rats were anesthetized with 3% pentobarbital sodium, the pre‐weighed filter paper was placed on the tail of the rat, and then cut off the tail with surgical scissors 3 cm from the root of the tail, and the pre‐weighed gauze, gelatin sponge, and cellulose aerogel were quickly placed in the tail of the rat, the amount of bleeding and the bleeding time were recorded, and the rat without hemostatic treatment was used as the control group.

For the rat liver hepatectomy hemorrhage model, rats were randomly divided into six groups (n = 3) and anesthetized with 3% pentobarbital sodium. Rats were promptly immobilized before fully exposing the liver. Pre‐weighed filter paper was positioned under the liver prior to excising a section of hepatic tissue. Gauze, gelatin sponges, and cellulose aerogels were rapidly administered to the bleeding liver injuries, while untreated rats served as controls. Hemorrhage volume and time were recorded. In addition, In the rat liver liver‐perforation hemorrhage model, the rats were randomly divided into 6 groups (n = 3), the rats were fixed on the operating table after anesthesia, the rat liver was fully exposed, the pre‐weighed filter paper was placed under the liver, a hole with a diameter of ≈8 mm was made above the liver, and the pre‐weighed gauze, gelatin sponge and cellulose aerogel were quickly placed above the trauma, and the rats without hemostatic treatment were used as the control group to record the amount of bleeding and bleeding time. The wound was sutured, rats were fed for 7 to 14 days, and liver was removed for 7 and 14 days and fixed with 4% paraformaldehyde for histological staining analysis.

For the rabbit cardiac hemorrhage model, rabbits were randomly divided into 6 groups and anesthetized with 3% pentobarbital sodium. Rabbits were immobilized to fully expose the heart. Myocardial puncture wounds were created using a 0.9 mm diameter needle. Pre‐weighed gauze, gelatin sponges, and cellulose aerogels were promptly administered to the bleeding sites, while untreated rabbits served as controls. Hemorrhage volume and time were documented. Finally, wounds were sutured closed, and rabbits were housed for another 7 or 14 days, then hearts were excised and fixed in 4% paraformaldehyde for histological analysis.

### Subcutaneous Implantation and Histological Observations

To assess in vivo biocompatibility, CPP aerogels, and gelfoam samples were sterilized prior to implantation. Rats were anesthetized and immobilized before making ≈1 cm dorsal incisions. Gelfoam and CPP aerogels (5 mm x 8 mm) were implanted into the wounds before closure with absorbable sutures. At 7, 14, 28, and 56 days post‐implantation, the tissues surrounding the materials were harvested and fixed in 4% paraformaldehyde for histological analysis. According to the standard protocol, the H&E staining, CD68 (1:300), and F4/80 (1:300) immunohistochemical staining was performed.

### Statistical Analysis

Results were presented as mean ± standard deviation based on at least three independent experiments. Data were analyzed using Origin software. One‐way ANOVA with post hoc tests was utilized for all statistical comparisons. P values <0.05 were considered statistically significant, * *p *<0.01, ** *p *<0.001, and **** *p *<0.0001.

## Conflict of Interest

The authors declare no conflict of interest.

## Supporting information

Supporting Information

## Data Availability

The data that support the findings of this study are available from the corresponding author upon reasonable request.
