# Supplementary material for: Carbonized Cellulose Aerogel Derived from Waste Pomelo Peel for Rapid Hemostasis of Trauma‐Induced Bleeding
Source: Adv Sci (Weinh). 2024 Mar 13;11(19):2307409. doi: 10.1002/advs.202307409 (PMC11109610; doi:10.1002/advs.202307409)
Supplement: Supplementary file 1 — Supporting Information [file ADVS-11-2307409-s001.pdf]

ADVANCED SCIENCE
Open Access

Supporting Information

for *Adv. Sci.*, DOI 10.1002/advs.202307409

Carbonized Cellulose Aerogel Derived from Waste Pomelo Peel for Rapid Hemostasis of Trauma-Induced Bleeding

Wenbing Wan, Yang Feng, Jiang Tan, Huiping Zeng, Rafeek Khan Jalaludeen, Xiaoxi Zeng, Bin Zheng, Jingchun Song, Xiyue Zhang, Shixuan Chen and Jingye Pan**

Supporting information

Carbonized Cellulose Aerogel Derived from Waste Pomelo Peel for Rapid Hemostasis of Trauma-Induced Bleeding

Wenbing Wan, Yang Feng, Jiang Tan, Huiping Zeng, Rafeek Khan Jalaludeen, Xiaoxi Zeng, Bin Zheng, Jingchun Song, Xiyue Zhang, Shixuan Chen*, Jingye Pan*

W. Wan, Yang Feng, Huiping Zeng, Rafeek Khan Jalaludeen

The Second Affiliated Hospital, Jiangxi Medical College, Nanchang University, Nanchang, Jiangxi, 330006 China.

J. Tan, J. Pan

Key Laboratory of Intelligent Treatment and Life Support for Critical Diseases of Zhejiang Province, Zhejiang Engineering Research Center for Hospital Emergency and Process Digitization, The First Affiliated Hospital of Wenzhou Medical University, Wenzhou, Zhejiang, 325000 China

E-mail: panjingye@wzhospital.cn

J. Tan, X. Zhang, S. Chen

Zhejiang Engineering Research Center for Tissue Repair Materials, Wenzhou Institute, University of Chinese Academy of Sciences, Wenzhou, Zhejiang, 325000 China

E-mail: chensx@ucas.ac.cn

X. Zhang

Macau University of Science and Technology, Taipa, Macau 999078, China

X. Zeng

Biomedical Big Data Center, West China Hospital, Sichuan University, Chengdu, China

B. Zheng

Wenzhou Safety (Emergency) Institute of Tianjin University, Wenzhou, China.

J. Song

Department of Critical Care Medicine, No. 908th Hospital of PLA Logistic Support Force, Nanchang 330002, China

W. Wan, Yang Feng, and J. Tan contributed equally to this work.

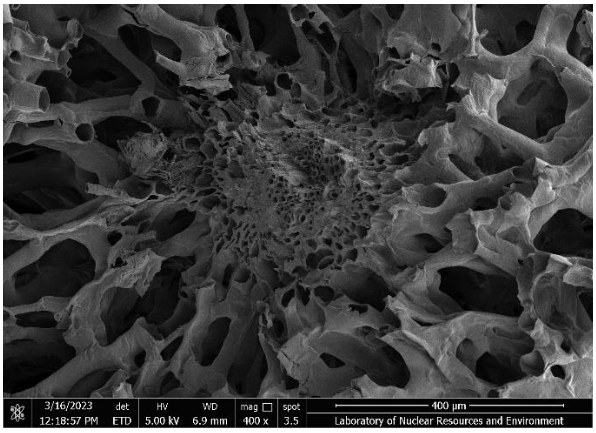

Figure S1. The internal structure of the original pomelo peel.

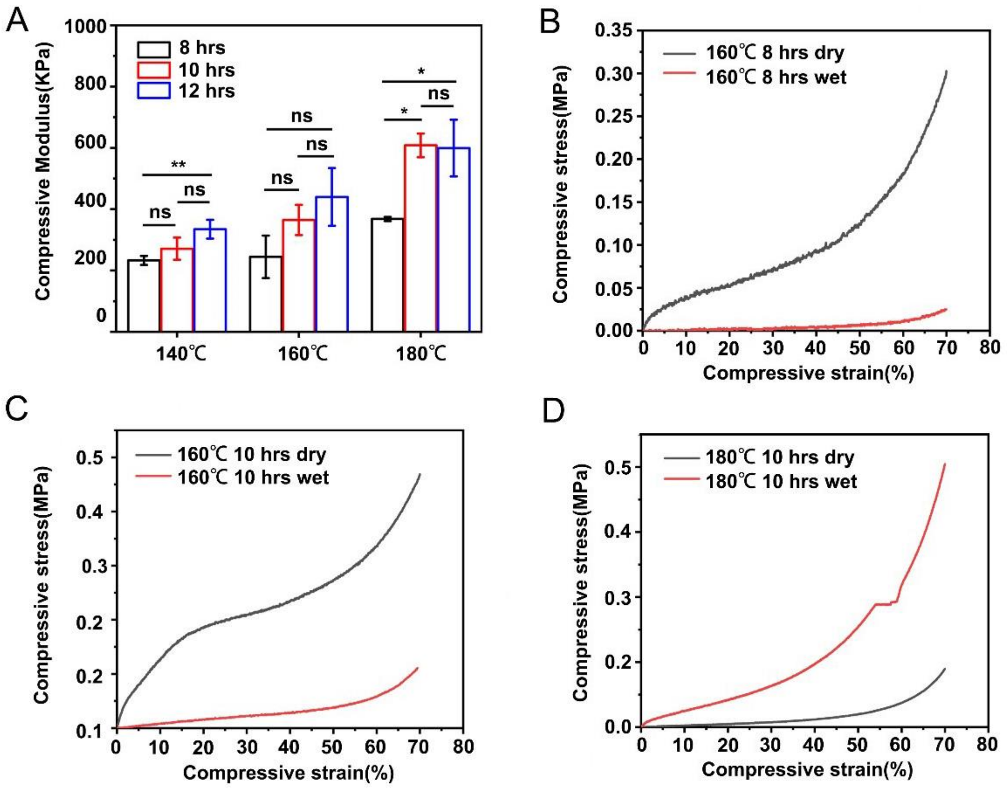

Figure S2. A) The compressive modules of different CPPs. B-D) The compression strain-stress curve of (160 °C, 8 hrs) CPP, (160 °C, 10 hrs) CPP, and (180 °C, 10 hrs) CPP under dry and wet conditions, respectively. *$p<0.05$, **$p<0.01$.

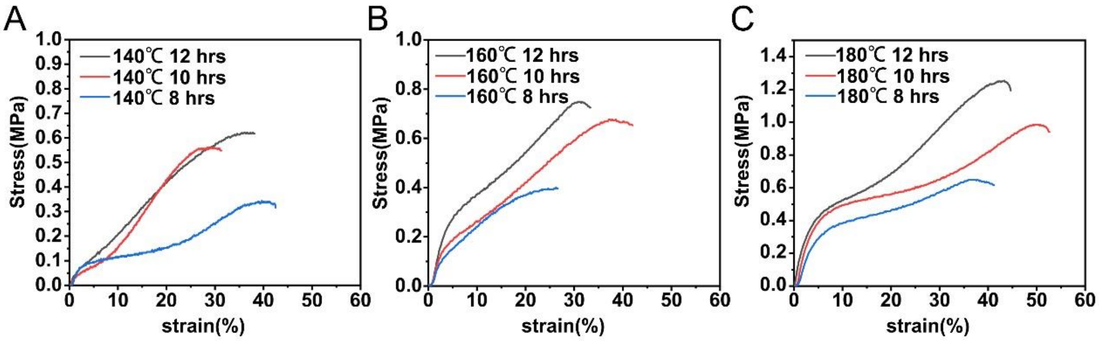

Figure S3. A) The tensile strain-stress curve of different CPPs that carbonized at 140 ℃ for 8, 10, and 12 hrs, respectively. B) The tensile strain-stress curve of different CPPs that carbonized at 160 ℃ for 8, 10, and 12 hrs, respectively. C) The tensile strain-stress curve of different CPPs that carbonized at 180 ℃ for 8, 10, and 12 hrs, respectively.

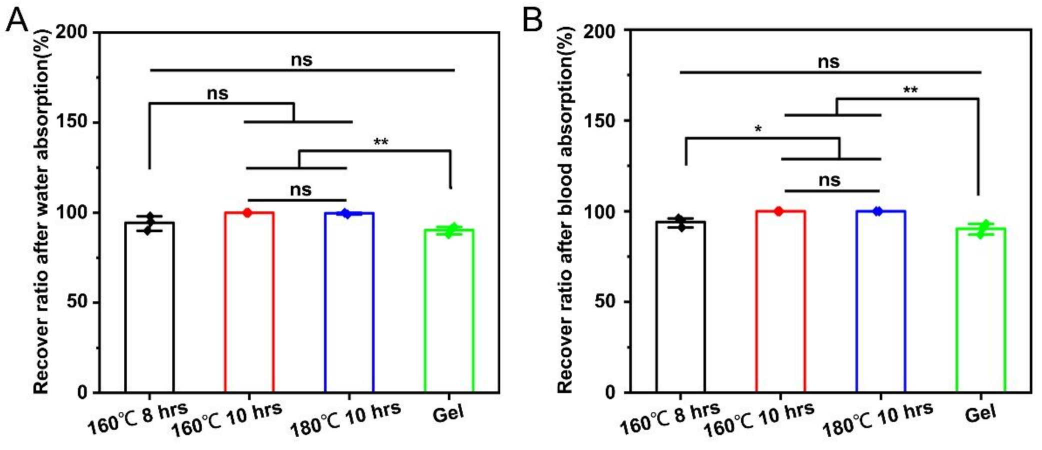

Figure S4. A) The shape recovery ratio of wet CPPs and gelfoam after compression. B) The shape recovery ratio of blood-absorb CPPs and gelfoam after compression.

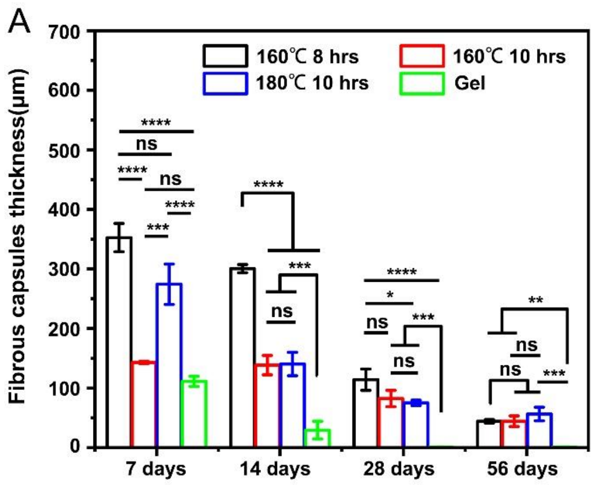

Figure S5. The thickness of formed fibrous capsules of different CPPs and gelfoam groups after 7, 14, 28, and 56 days of subcutaneous implantation. *p<0.05, **p<0.01, ***p<0.001, ****p<0.0001.

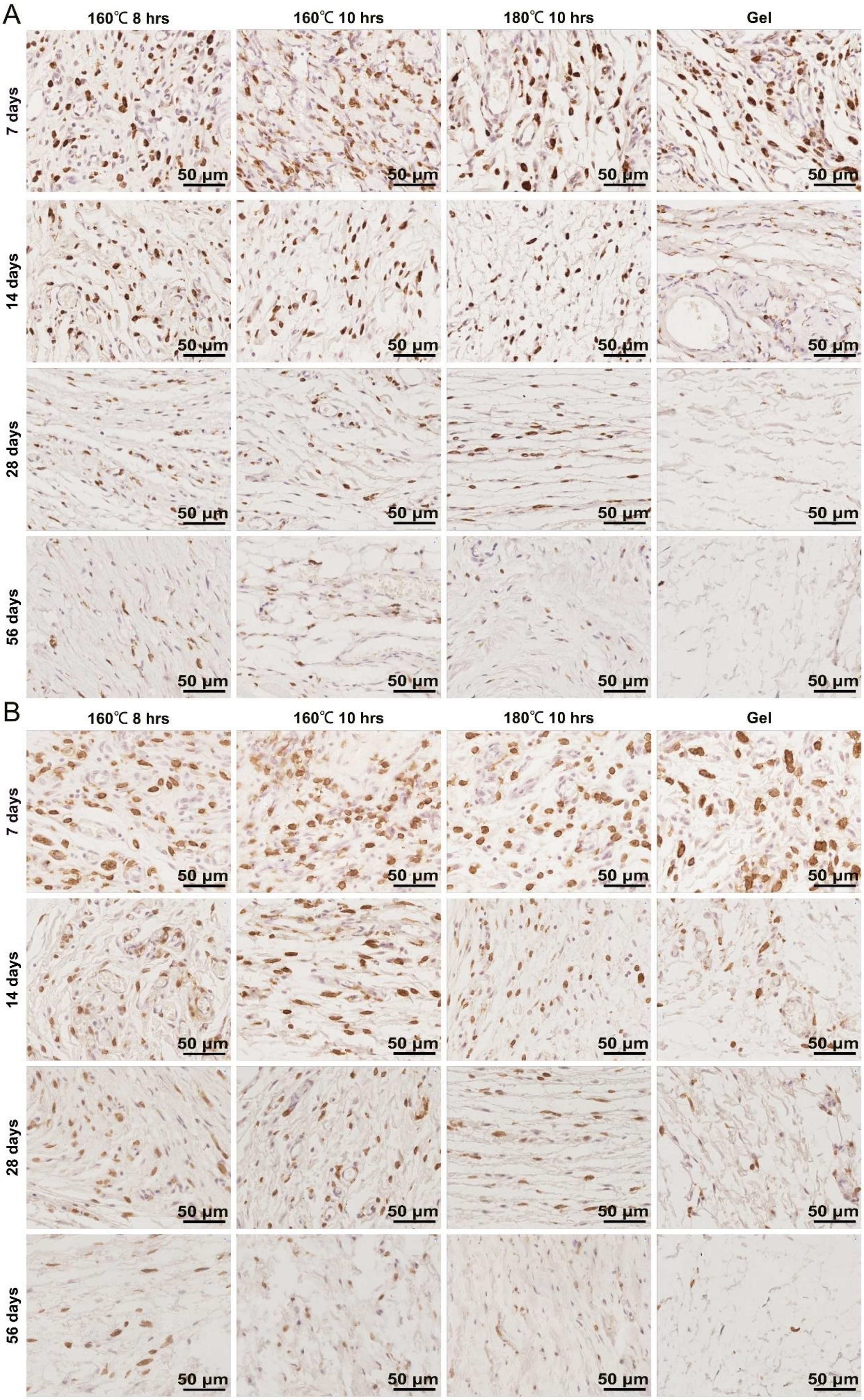

A
160℃ 8 hrs
160℃ 10 hrs
180℃ 10 hrs
Gel
7 days
14 days
28 days
56 days
50 μm
B
160℃ 8 hrs
160℃ 10 hrs
180℃ 10 hrs
Gel
7 days
14 days
28 days
56 days
50 μm

Figure S6. The expression of CD68 (A) and F4/80 (B) in the fibrous capsule area of different CPPs and gelfoam groups after 7, 14, 28, and 56 days of subcutaneous implantation.

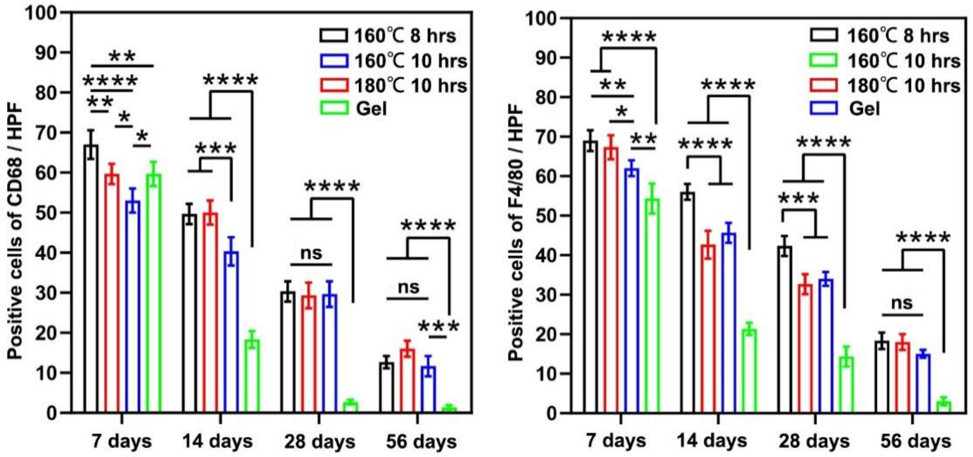

Figure S7. The quantification of CD68 and F4/80 positive cells of different CPPs and gelfoam groups after 7, 14, 28, and 56 days of subcutaneous implantation.
